# Fish, Fish Oils and Cardioprotection: Promise or Fish Tale?

**DOI:** 10.3390/ijms19123703

**Published:** 2018-11-22

**Authors:** Akshay Goel, Naga Venkata Pothineni, Mayank Singhal, Hakan Paydak, Tom Saldeen, Jawahar L. Mehta

**Affiliations:** 1 Division of Cardiology, University of Arkansas for Medical Sciences and Central Arkansas Veterans Healthcare System, Little Rock, AR 72205, USA; AGoel@uams.edu (A.G.); NVPothineni@uams.edu (N.V.P.); HPaydak@uams.edu (H.P.); tom.saldeen@uppsalapharma.se (T.S.); 2 Cape Fear Valley Hospital, Fayetteville, NC 28304, USA; drmayanksinghal@gmail.com

**Keywords:** fish oil, omega-3 fatty acids, eicosapentaenoic acid (EPA), docosahexaenoic acid (DHA), cardiovascular disease

## Abstract

Fish and commercially available fish oil preparations are rich sources of long-chain omega-3 polyunsaturated fatty acids. Eicosapentaenoic acid (EPA) and docosahexaenoic acid (DHA) are the most important fatty acids in fish oil. Following dietary intake, these fatty acids get incorporated into the cell membrane phospholipids throughout the body, especially in the heart and brain. They play an important role in early brain development during infancy, and have also been shown to be of benefit in dementia, depression, and other neuropsychiatric disorders. Early epidemiologic studies show an inverse relationship between fish consumption and the risk of coronary heart disease. This led to the identification of the cardioprotective role of these marine-derived fatty acids. Many experimental studies and some clinical trials have documented the benefits of fish oil supplementation in decreasing the incidence and progression of atherosclerosis, myocardial infarction, heart failure, arrhythmias, and stroke. Possible mechanisms include reduction in triglycerides, alteration in membrane fluidity, modulation of cardiac ion channels, and anti-inflammatory, anti-thrombotic, and anti-arrhythmic effects. Fish oil supplements are generally safe, and the risk of toxicity with methylmercury, an environmental toxin found in fish, is minimal. Current guidelines recommend the consumption of either one to two servings of oily fish per week or daily fish oil supplements (around 1 g of omega-3 polyunsaturated fatty acids per day) in adults. However, recent large-scale studies have failed to demonstrate any benefit of fish oil supplements on cardiovascular outcomes and mortality. Here, we review the different trials that evaluated the role of fish oil in cardiovascular diseases.

## 1. Introduction

Cardiovascular disease (CVD) is a leading cause of death in the United States [1]. However, despite extensive advances in our knowledge of nutritional options in the prevention and therapy of CVD, the benefits of dietary fish and fish oil supplementation on CVD remains debatable. Fish oil is a rich source of long-chain omega-3 (ω-3) polyunsaturated fatty acids (PUFAs)—eicosapentaenoic acid (EPA), and docosahexaenoic acid (DHA). The possible cardioprotective role of fish consumption was first identified in the early studies of Greenland’s Inuit population who were found to have a low incidence of myocardial infarction (MI) compared to their Danish counterparts. The benefit was attributed to the high fish consumption by the Inuits [2,3]. This was followed by nearly four decades of research, including animal studies, epidemiological studies, randomized controlled trials (RCTs), meta-analyses of epidemiological cohort studies, and trial meta-analyses. Many of these studies demonstrated the cardioprotective effects of fish consumption and fish oil supplementation [4,5,6,7,8,9,10]. The rising amount of evidence led to recommendations regarding consumption of seafood/fish and/or their dietary supplementation with ω-3 PUFA from the Food and Drug Administration (FDA) [11]. In 2012, a prescription fish oil formulation was approved by the US FDA. Fish oil continues to be the most popular natural supplement in the US, used by nearly 18.8 million adults [12]. However, several recent large-scale studies have failed to demonstrate any significant benefits of fish oil supplements on cardiovascular outcomes and mortality [13,14,15,16,17]. In this review, we discuss the structure and metabolism of marine-derived ω-3 PUFAs, their proposed benefits and molecular mechanisms of action, and the evidence regarding their role in CVD. We conclude by providing possible reasons for the conflicting evidence and recommendations regarding the dietary intake of fish and fish oil supplements.

## 2. Structure and Metabolism of PUFAs

Fatty acids are long-chain hydrocarbons with a carboxylic acid group at one end (alpha terminal) and methyl group at the other end (omega terminal). They can be classified based on the number of double bonds in their side chains—saturated fatty acids (no double bond), monounsaturated fatty acids or MUFAs (single double bond), and polyunsaturated fatty acids or PUFAs (two or more double bonds). PUFAs can be classified further by the length of the carbon chain and the position of the first double bond from the methyl terminal into omega-6 (ω-6 or n-6) or omega-3 (ω-3 or n-3). For example, linoleic acid (LA) or 9,12-octadecadienoic acid (C_18:2_) has 18 carbon atoms with 2 double bonds. Since the first double bond from the methyl terminal is at the sixth position, it is an ω-6 PUFA. Similarly, alpha-linolenic acid (ALA) is 9,12,15-octadecatrienoic acid (C_18:3_), meaning it has 18 carbon atoms with 3 double bonds. However, in this case, the first double bond is at the third position from the methyl terminal, and hence it is an ω-3 PUFA. Both LA and ALA are considered essential fatty acids since they cannot be synthesized by humans and must be ingested via their diet.

The essential fatty acids LA and ALA are then metabolized to other fatty acids through desaturase and elongase enzymes. LA (ω-6) is metabolized to arachidonic acid (5,8,11,14-eicosatetraenoic acid, C_20:4_, ω-6). Similarly, ALA (ω-3) is converted to EPA (5,8,11,14,17-eicosapentaenoic acid, C_20:5_, ω-3) and DHA (4,7,10,13,16,19-docosahexaenoic acid, C_22:6_, ω-3). Thus, EPA and DHA are traditionally considered non-essential since, technically speaking, they can be synthesized from ALA. However, this pathway is slow and inefficient [18]. Therefore, for all practical purposes, the dietary intake of EPA and DHA is “essential” and crucial to obtain health benefits.

After absorption from the intestine as chylomicrons, fatty acids are transported to the liver and other tissues. PUFAs are subsequently incorporated into the phospholipid bilayer of plasma membranes, and affect membrane fluidity and signaling. ω-6 and ω-3 PUFAs have opposite effects in the body. Diets rich in ω-6 are precursors of eicosanoids associated with inflammation, vasoconstriction, and platelet aggregation [19]. Acute self-limited inflammation is a protective response to infection and injury. However, excessive inappropriate inflammation has been linked to atherosclerosis and cancer. On the other hand, ω-3 PUFAs are precursors of anti-inflammatory molecules and provide benefits against chronic inflammatory conditions, like diabetes, ischemic heart disease, and cancer [20]. These molecular mechanisms are discussed in further detail in a later section of this review.

**Figure 1:**
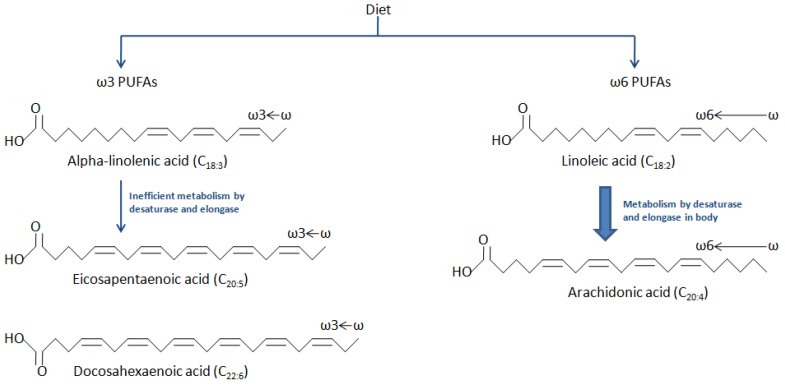
Structure and metabolism of major polyunsaturated fatty acids (PUFAs).

The structure and metabolism of major PUFAs is depicted in Figure 1.

## 3. Dietary Sources of Major PUFAs

Most vegetable oils and crop seeds, like corn, sunflower, soybean, and canola oils, are a rich source of ω-6 LA with lesser amounts of ω-3 ALA. On the other hand, flax, walnuts, and chia seeds are a rich source of ALA, as are some green leafy vegetables.

Fatty fish and other seafood are the most important dietary sources of EPA and DHA. Wild (marine) fish feed on phyto- and zoo-planktons, and are therefore a richer source of EPA and DHA than cultivated (farmed) fish which feed on cereals and vegetable oils [20]. Cod liver and algal oil have been proposed to be non-traditional marine sources of EPA and DHA. Finally, marine-derived ω-3 fortified food products, like cereals, pastas, dairy products, eggs, meat, salad dressings, and oils are now available. They are a potential option for vegetarians and those who dislike seafood [18]. The principal dietary sources of PUFAs are shown in Table 1 [21,22].

## 4. Proposed Cardioprotective Benefits of ω-3 PUFAs and Their Molecular Mechanisms of Action

Fish and fish oil consumption has been shown to decrease cardiovascular events and mortality in many secondary prevention trials that included high-risk patients with recent myocardial infarction or heart failure. Several molecular mechanisms for this cardioprotective effect of ω-3 PUFAs have been proposed.

### 4.1. Anti-Inflammatory Effect

ω-3 PUFAs have pleiotropic anti-inflammatory effects. They readily replace arachidonic acid in cell membranes. This results in decreased production of ω-6 arachidonic acid-derived inflammatory mediators, like prostaglandin (PG) E_2_, thromboxane (TX) A_2_, and leukotrienes (LTs) A_4_, B_4_, C_4_, D_4_, and E_4_ [23,24]. ω-3 PUFAs generate less potent inflammatory substrates, like 3-series PGs and TXs and 5-series LTs [24]. 3-series PGs have less potent biological effects (compared to 2-series PGs) and TX A_3_ lacks pro-platelet aggregatory properties. 5-series LTs are relatively less effective as pro-inflammatory molecules. EPA and DHA are metabolized to anti-inflammatory mediators, like resolvins, protectins, and the G protein-coupled receptor 120 [24,25]. Additionally, cell membranes with higher ω-3: ω-6 PUFA ratio have higher fluidity. Marine-derived ω-3 PUFAs have been shown to reduce the circulating levels of pro-inflammatory cytokines like interleukin (IL)-1, IL-6, and the tumor necrosis factor (TNF)-*α* [26]. ω-3 PUFAs also regulate intracellular signaling pathways to inactivate nuclear transcriptional factors. They decrease expression of inflammatory genes via the downregulation of nuclear factor (NF)-κB. The inhibition of NF-κB is mediated by the activation of peroxisome proliferator-activated receptors (PPAR) [27].

### 4.2. Improved Endothelial Function

Endothelial dysfunction from loss of endothelial-derived nitric oxide (NO) synthesis results in predisposition to accelerated atherosclerosis and adverse vascular events. Marine-derived ω-3 PUFAs cause translocation and activation of endothelial NO synthase (eNOS) into the cytosol, resulting in vasodilation and improved endothelial function [25,28]. Recently, EPA was shown to prevent saturated fatty acid-induced vascular endothelial dysfunction through regulation of long-chain acyl-coA synthetase expression [29]. Endothelial function is additionally improved by reduced expression of endothelial vascular cell adhesion molecules, resulting in attenuated leukocyte adhesion to endothelium [30,31].

### 4.3. Atherosclerotic Plaque Stabilization

EPA and DHA inhibit the proliferation and migration of smooth muscle cells (SMCs), a central step in atherosclerotic plaque formation and progression. Vasa vasorum is a network of microvessels extending up to the plaque base, which is vital for plaque progression. ω-3 PUFAs interfere with the neovascularization of vasa vasorum, thereby suppressing plaque development [25].

Apart from reducing plaque progression, ω-3 PUFAs also contribute to plaque stability. Plaque vulnerability predisposes people to plaque erosion or rupture, which causes acute coronary syndrome. High tissue levels of EPA and DHA decrease macrophage infiltration and the release of matrix metalloproteinases (MMPs), resulting in greater plaque stability [23]. The addition of EPA to statin therapy has been shown to reduce the lipid core in coronary plaques [32]. Plaques in patients receiving fish oil are more likely to be fibrous cap atheromas, with fewer macrophages and lesser inflammation, and are therefore more likely to be stable [33].

### 4.4. Effect on Lipid Metabolism

ω-3 PUFAs modulate the activity of genes that control lipid homeostasis. Large doses of fish oil interfere with the synthesis of very low-density lipoprotein (VLDL) via inhibition of sterol receptor element-binding protein-1c. This results in a marked lowering of serum TG levels [23]. Although ω-3 fatty acids do not affect the serum levels of total cholesterol and low-density lipoprotein (LDL), fish oil has been shown to reduce remnant lipoproteins and post-prandial lipemia after fatty meals [30,31]. Remnant lipoprotein levels and post-prandial lipemia are involved in the pathogenesis of sudden cardiac death (SCD) [23]. Marine-derived ω-3 fatty acids also cause a favorable change in high-density lipoprotein (HDL) by increasing the large, cholesterol-rich HDL2 fraction and lowering the small, TG-rich HDL3 fraction [30].

### 4.5. Anti-Thrombotic Effect

EPA inhibits the synthesis of platelet TXA_2_ which causes platelet aggregation and vasoconstriction. Both EPA and DHA antagonize the TXA_2_ and PGH_2_ receptors in human platelets [34]. There are reports that ω-3 PUFA reduce fibrinogen levels and increase tissue plasminogen-activator concentrations [30,31,35].

### 4.6. Anti-Arrhythmic Effect

ω-3 PUFAs modulate the activity of multiple ion channels and stabilize the cardiomyocyte membrane, thereby preventing tachyarrhythmias and SCD [5]. EPA and DHA inhibit the voltage-gated sodium channels in cardiac myocytes, increase the voltage threshold for depolarization, and prolong the refractory period. They also modulate certain calcium channels, decrease free cytosolic calcium, and reduce membrane excitability further [24]. Part of the anti-arrhythmic action of these fatty acids is also due to their autonomic effects, like increased vagal tone [36]. In addition, low serum levels of EPA and DHA have been found to increase the risk of cardiogenic syncope in patients with Brugada syndrome [37].

### 4.7. Cardiac Remodeling

The OMEGA-REMODEL trial reported the beneficial effects of high-dose ω-3 PUFA therapy on cardiac remodeling in patients with MI. Cardiac magnetic resonance confirmed reduced ventricular remodeling and myocardial fibrosis after PUFA supplementation [38]. Similar findings of the preferential effects of ω-3 PUFAs on cardiac remodeling and heart failure were also seen in the Gruppo Italiano per lo Studio della Streptochinasi nell’Infarto Miocardico-Heart Failure (GISSI-HF) trial [10].

Cardiac myofibroblasts, activated by inflammatory signals or pressure overload, cause cardiac remodeling. Recently, a novel EPA metabolite called 18-hydroxy eicosapentaenoic acid (18-HEPE) has been identified, which prevents cardiac remodeling under pressure overload [39]. Higher dietary intake of EPA ethyl ester increases the plasma concentration of 18-HEPE, and may have beneficial long-term effects by preventing cardiac fibrosis [24].

Moreover, EPA and DHA improve cardiac mitochondrial function by increasing the efficiency of adenosine triphosphate (ATP) production [40].

### 4.8. Improved Exercise Tolerance

Decreased exercise capacity is a known risk factor for CVD. Recently, ω-3 PUFAs have been shown to improve exercise tolerance. This may be due to favorable effects on erythrocyte rheology and skeletal muscle function [25,41]. Improved exercise capacity may contribute to a lower risk of adverse cardiac events.

### 4.9. Improved Cognitive Function

Poor cognitive function is a risk factor for cardiovascular events [42]. Serum EPA levels have been shown to be independently associated with cognitive function in CVD patients [43]. Thus, improved dietary ω-3 PUFA intake might conceivably improve cognition and decrease the risk of cardiovascular events [25].

**Figure 2:**
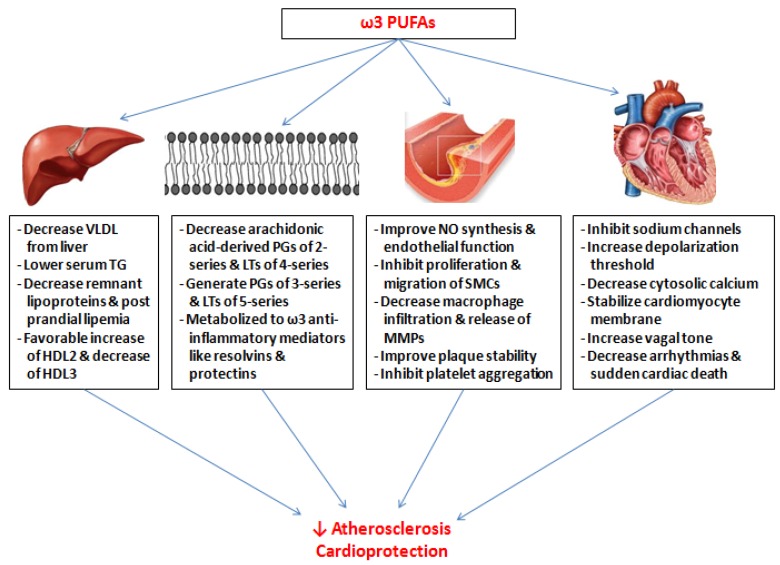
Pleiotropic cardioprotective effects of ω-3 PUFAs.

Figure 2 summarizes the various molecular mechanisms by which ω-3 PUFAs, like EPA and DHA, have been proposed to exert their cardioprotective effects.

## 5. Evidence from Trials and Meta-Analyses

The Diet and Reinfarction Trial (DART) was the first RCT to assess the benefits of dietary fish and fish oil in the secondary prevention of MI [4]. A total of 2033 patients with recent MI were followed for two years. Consumption of at least two servings (200–400 g) of fish per week was associated with a reduction in all-cause mortality. Similar beneficial effects were also noticed in participants who took fish oil capsules instead of fish.

The GISSI-Prevenzione trial included 11,324 patients with previous MI (within three months) [6]. The intervention group received one fish oil capsule per day, in addition to standard care. The intervention group was noted to have a 41% reduction in all-cause mortality and 53% reduction in SCD as early as four months into the study. The difference in all-cause mortality remained significant after a follow-up of 3.5 years, and was proposed to be primarily due to the anti-arrhythmic effects of EPA and DHA.

The DART-2 was a secondary prevention study conducted in men with stable angina (and not previous MI, unlike the first DART study) [44]. Patients who were advised to consume two portions of oily fish every week or take three fish oil capsules daily had a higher risk of cardiac mortality and SCD. Subgroup analysis revealed that this difference was driven by the fish oil capsule group. The study was largely criticized for its poor design and lack of blinding.

The Japan EPA Lipid Intervention Study (JELIS), a combined primary (14,981 patients) and secondary intervention (3664 patients) trial, included a total of 18,645 patients [9]. The EPA (1.8 g daily) plus statin group showed a reduction in major coronary events compared to the statin-alone group. Subgroup analysis revealed that the significant reduction in coronary events was mainly seen in patients with a history of coronary artery disease, and there was no benefit of EPA in primary prevention. However, the results of this study may have been diluted, as the consumption of fish in the Japanese population is high at baseline.

The GISSI-HF trial was designed to study the effects of daily EPA and DHA supplementation in patients with HF [10]. The ω-3 PUFA group had a reduction in all-cause mortality and cardiovascular hospitalizations compared to the control group.

The Alpha Omega trial was a secondary prevention study using 4837 patients with prior MI [14]. Low-dose EPA and DHA in margarine were given daily to the intervention group, while the control group received only plain margarine. There was no difference in cardiovascular events between the two groups. The lack of benefit from EPA and DHA in the Alpha Omega study [14], compared to the GISSI Prevenzione [6] and JELIS [9] trials (all secondary prevention trials in prior MI patients), may have been, in part, due to the lower treatment dose of the intervention arm.

The OMEGA study included German patients with MI in the two weeks prior to enrolment [13]. The treatment group received EPA and DHA daily, versus the control group, which received olive oil. After a follow-up of 1 year, no significant difference was found in the rates of adverse cardiovascular events, SCD, and all-cause mortality between the two groups. However, the study was concluded to be underpowered due to the lower-than-expected event rates and overestimation of the effect of ω-3 PUFAs.

SU.FOL.OM3 was a randomized trial evaluating the effects of B-vitamin and ω-3 PUFA supplementation in 2501 French patients with a recent acute coronary or cerebral ischemic event [15]. ω-3 PUFA intake did not significantly affect the major cardiovascular event rate. Like the OMEGA study, the event rate was lower than anticipated and so the trial was underpowered.

The Outcome Reduction with an Initial Glargine Intervention (ORIGIN) trial was the first study to investigate the effects of ω-3 PUFA supplementation on cardiovascular events in patients with pre-diabetes and diabetes [16]. Analysis of 12,536 patients over 6.2 years did not reveal any benefits of ω-3 PUFAs in reducing cardiovascular death, compared to the olive oil given to the control group. These results were in contrast to the findings of the GISSI Prevenzione [6] and GISSI-HF [10] trials. Inter-trial differences in patient baseline characteristics and concomitant therapies could be a possible explanation.

The Risk and Prevention study investigated the efficacy of ω-3 PUFAs in Italian patients with high risk of CVD but without previous MI [17]. After a follow-up of five years, there was no difference in the primary endpoint of cardiovascular death and hospitalization between the ω-3 PUFA treatment group and olive oil control group. However, subgroup analysis revealed that the ω-3 PUFA group had fewer heart failure hospitalizations. Additionally, women in the treatment group had lower rates of primary endpoint than the control group. The results of this study may not be generalizable due to the Mediterranean dietary habits in the Italian population.

The PREDIMED trial in 7447 patients reported that a Mediterranean diet, with 50 g of extra-virgin olive oil daily, significantly reduced cardiovascular events and death at a follow-up period of 4.8 years [45]. It remains unknown whether the benefit was due to the Mediterranean diet alone, olive oil alone, or whether it was a combined effect. The OMEGA [13], ORIGIN [16], and Risk and Prevention [17] trials did not detect a significant difference between the overall outcomes in the ω-3 PUFA group and olive oil control group. However, the results of the PREDIMED trial [45] indicate that olive oil itself may have some cardioprotective benefits and is likely not an ideal control.

The Age-Related Eye Disease Study 2 (AREDS2) trial included 4203 patients at high risk of CVD and either intermediate or advanced macular degeneration [46]. Patients were randomly grouped to the ω-3 PUFA group (650 mg EPA plus 350 mg DHA), macular xanthophyll group (10 mg lutein plus 2 mg zeaxanthin), combination therapy, or matching placebos. Long-chain ω-3 PUFAs or macular xanthophylls did not reduce the risk of CVD events.

Rizos et al. performed a meta-analysis of 20 RCTs including 68,680 patients to study the role of ω-3 PUFA supplementation on major cardiovascular outcomes [47]. Overall, ω-3 PUFAs were not associated with cardiovascular benefits.

Studies have also evaluated the role of ω-3 PUFAs in the secondary prevention of atrial fibrillation [48,49]. The FORWARD trial included 586 participants with previous atrial fibrillation who were randomized to receive either ω-3 PUFA for 1 g per day, or a placebo for one year [48]. PUFA supplementation did not reduce recurrent atrial fibrillation. Mariani et al. performed a meta-analysis of 16 trials covering 4677 patients and concluded that ω-3 PUFAs have no effect in preventing recurrent or post-operative atrial fibrillation [49].

The results of the ASCEND trial were recently published [50]. It was a randomized, placebo-controlled, blinded trial in 15,480 patients followed for 7.4 years. The study aimed to assess the efficacy and safety of taking 100 mg of aspirin daily in preventing cardiovascular events and cancer in diabetic patients without known CVD. This study also investigated whether daily ω-3 PUFA supplementation decreased cardiovascular events in this population. Aspirin use was noted to prevent cardiovascular events in patients, but also caused major bleeding events. Compared to the placebo group that received olive oil capsules, 1 g of ω-3 PUFA supplementation daily failed to decrease the risk of serious vascular events in diabetics without known CVD.

The Reduction of Cardiovascular Events with EPA-Intervention Trial (REDUCE-IT) was recently concluded [51]. The study involved 8179 high-risk patients with hypertriglyceridemia on statin therapy who were randomized to receive either 4 g of ethyl EPA daily, or a placebo. After a median follow-up of 4.9 years, there was an approximately 25% reduction in the risk of major adverse cardiovascular events in the treatment group.

The Vitamin D and Omega-3 Trial (VITAL) results were recently announced [52]. The study included 25,871 participants with an objective to assess the effect of daily vitamin D_3_ (2000 IU) and fish oil supplement (1 g) in the primary prevention of cancer and CVD. After a follow-up of over five years, ω-3 PUFA supplementation did not result in a lower incidence of cardiovascular events or cancer, compared to the placebo.

The STRENGTH study is an ongoing RCT that will enroll approximately 13,000 patients with hypertriglyceridemia, low HDL, and a high risk for CVD [53]. Patients are being randomized to receive either statin with corn oil or statin with prescription ω-3 carboxylic acids. The study is anticipated to be completed in 2019.

Results of various published trials and meta-analyses discussed above are presented in Table 2.

## 6. Available ω-3 PUFA Formulations

ω-3 PUFA ethyl esters was the first formulation approved by the FDA in the year 2004. It was marketed under the trade name Omacor^®^ by Reliant Pharmaceuticals and approved for use in patients with serum triglyceride levels greater than 500 mg/dL. It was later renamed to Lovaza^®^ (GlaxoSmithKline). Ethyl EPA or icosapent ethyl, marketed under the name Vascepa^®^ by Amarin Pharmaceuticals, was approved in 2012. It differed from the earlier formulation in that it contained only ethyl esters of EPA without any DHA [54].

In 2014, ω-3 carboxylic acids, marketed under the name Epanova^®^ by AstraZeneca, was approved for hypertriglyceridemia greater than 500 mg/dL. This formulation consists of free fatty acids instead of the prodrug, and therefore does not require pancreatic lipase for conversion to active form. Thus, it can be taken independent of meals with good bioavailability [54]. The ECLIPSE [55] and ECLIPSE II [56] studies compared the pharmacokinetics of ethyl esters and carboxylic acids. Both studies reported much higher bioavailability with the carboxylic acid formulations.

Despite newer options, ethyl esters continue to be the most commonly prescribed due to its generic options.

Differences between various ω-3 prescription products are shown in Table 3 [22].

## 7. Side-Effects and Safety Concerns

Fish oil supplements are generally well tolerated. The most common side effects are gastrointestinal, like nausea, eructation, and diarrhea. Certain large fish (like king mackerel, shark, swordfish) have a higher chance of being contaminated with methyl mercury and therefore should be avoided by pregnant/breastfeeding women and children. There have been some concerns about an increased risk of minor bleeding with ω-3 fatty acid supplementation, especially in patients taking aspirin and statins. However, no major bleeding events have been reported in trials to date.

## 8. Conclusions

Data from early epidemiologic and observational studies have shown there to be cardioprotective benefits from fish and fish oil consumption. However, most primary prevention trials and recent secondary prevention trials have failed to replicate similar results. Several possibilities could explain the difference in results—the high efficacy of modern-day pharmacotherapy (like statins) and revascularization that attenuates the benefit of ω-3 fatty acids, a lower dose of EPA and DHA supplementation in trials than what was needed, insufficient length of follow-up to see benefits, an improved diet with a higher consumption of fish and other seafood which may account for the decreased magnitude of benefits from fish oil capsules over time, improper study design with use of olive oil as a control (olive oil itself has cardioprotective properties making it an unideal control), and fewer-than-anticipated events leading to underpowered studies. Some trials with a large sample size and strong study design are ongoing, and may shed useful light on the subject.

Based on the current evidence, individuals are advised to consume a healthy diet with two servings of fatty fish every week. Such a food-based approach also supplies several other beneficial nutrients apart from ω-3 PUFAs. For those who cannot consume fish, fish oil supplements containing EPA and DHA have a good safety profile and may be reasonable options, especially in patients with pre-existing CVD, heart failure, and hypertriglyceridemia.

## Figures and Tables

**Table 1:** Dietary sources of major PUFAs.

PUFA	Dietary Source
Linoleic acid (ω-6)	Corn, safflower, soybean, sunflower oils
Alpha-linolenic acid (plant-derived ω-3)	Flaxseed oil, canola (rapeseed) oil, walnuts, seeds of chia, perilla, green leafy vegetables
Eicosapentaenoic and Docosahexaenoic acids (marine-derived ω-3)	Fish, fish oil, other seafood, beef, lamb, ω-3 fortified foods

**Table 2:** Trials and meta-analyses of ω-3 PUFAs in cardiovascular disease (CVD).

Study	Study Design	Number of Patients	Intervention	Follow-Up	Outcome
DART [4] 1989	Secondary prevention RCT	2033	200–400 g fish per week	2 years	29% reduction in mortality
GISSI Prevenzione [6] 1999	Secondary prevention RCT	11,324	882 mg EPA and DHA daily	3.5 years	15–20% reduction in mortality and CV events
DART-2 [44] 2003	Secondary prevention RCT	3114	2 fish servings per week or 3 fish oil capsules daily	3-9 years	Higher cardiac mortality and SCD
JELIS [9] 2007	Primary and secondary prevention RCT	18,645	1.8 g EPA daily	4.6 years	19% reduction in coronary events in CAD patients, no benefit in primary prevention
GISSI-HF [10] 2008	Secondary prevention RCT	6975	840 mg EPA and DHA daily	3.9 years	9% reduction in mortality and 8% reduction in hospitalizations
Alpha Omega [14] 2010	Secondary prevention RCT	4837	226 mg EPA and 150 mg DHA daily	3.4 years	No benefit
OMEGA [13] 2010	Secondary prevention RCT	3851	460 mg EPA and 380 mg DHA daily (vs olive oil control)	1 year	No benefit
SU.FOL.OM3 [15] 2010	Secondary prevention RCT	2501	600 mg EPA and DHA daily	4.7 years	No benefit
ORIGIN [16] 2012	Secondary prevention RCT	12,536	465 mg EPA and 375 mg DHA daily (vs olive oil control)	6.2 years	No benefit
Rizos et al. [47] 2012	Meta-analysis of 20 RCTs	68,680	1000 mg EPA and DHA daily (median)	-	No benefit
Risk and Prevention [17] 2013	Primary prevention RCT	12,513	850 mg of EPA and DHA daily (vs olive oil control)	5 years	No benefit
AREDS2 [46] 2014	Primary prevention RCT	4203	650 mg EPA plus 350 mg DHA daily	4.8 years	No benefit
ASCEND [50] 2018	Primary prevention RCT	15,480	1 g ω-3 PUFA daily (vs olive oil control)	7.4 years	No benefit
REDUCE-IT [51] 2018	Primary prevention RCT	8179	4 g ethyl EPA daily	4.9 years	25% reduction in major CV events
VITAL [52] 2018	Primary prevention RCT	25,871	1 g ω-3 PUFA daily	5.3 years	No benefit

**Table 3:** ω-3 PUFA formulations.

	Ethyl Esters of EPA and DHA	Ethyl Esters of EPA Only	Free Fatty Acids of EPA and DHA
Brand name	Lovaza^®^ (GlaxoSmithKline)	Vascepa^®^ (Amarin Pharmaceuticals)	Epanova^®^ (AstraZeneca)
Approval date	2004	2012	2014
EPA/DHA (g per capsule)	EPA 0.465 g DHA0.375 g	EPA 1 g	EPA 0.550 g DHA 0.200 g
Dosing	2 g (2 capsules) twice daily or 4 g (4 capsules)once daily WITH MEALS	2 g (2 capsules) twice daily WITH MEALS	2 g (2 capsules)or 4 g (4 capsules) once daily WITH OR WITHOUT MEALS
